# E-mental Health in the Age of AI: Data Safety, Privacy Regulations and Recommendations

**DOI:** 10.31083/AP44279

**Published:** 2025-06-26

**Authors:** Hanwen Zhang, Yanna Mao, Yibin Lin, Dexing Zhang

**Affiliations:** ^1^JC School of Public Health and Primary Care, The Chinese University of Hong Kong, Hong Kong, China; ^2^School of Health Management, Southern Medical University, 510091 Guangzhou, Guangdong, China; ^3^School of Nursing, The Hong Kong Polytechnic University, Hong Kong, China

## 1. Mental Health Problems Are a Global Challenge

Mental health problems pose a major public health challenge globally. It has been reported that about 14% of the world’s population experienced mental disorders, and 17% of the total years lived with disability were attributable to these disorders in 2021 [1]. An increasing trend of mental health issues has been observed among younger generations; in 2020, depressive and anxiety disorders were most prevalent in the 20–35-year-old age group. Significant unmet needs for mental health treatment persist [2, 3], due to underscreening, underdiagnosis, and undertreatment. These issues are compounded by stigma, a lack of public awareness and literacy, and a healthcare system that suffers from insufficient mental health manpower, resources, and access.

## 2. The Role of E-mental Health (EMH) in Mitigating Growing Global Challenges 

EMH interventions have been applied in primary care settings, hospitals, long-term care facilities, and non-clinical settings such as home or community. It showed effectiveness in treating anxiety and depression, eating disorder, work-related stress etc. [4, 5, 6, 7], especially in rural area where residents have lower health literacy and limited access to healthcare [8]. Virtual mental health evaluation services has improved patient engagement and continuity of care following hospital discharge, as well as the efficiency in hospital emergency department [9].

EMH treatments yield positive and sustaining effects and remain stable post-treatment across various patient groups and therapy types [10]. There is a growing availability of patient-driven, web-based resources for depression and other chronic care managements in primary care intervention [11]. Digital phenotyping (DP) has been widely integrated into EMH interventions including heart rate estimation, exercise/physical activity monitoring, and sleep tracking, supported by validated effectiveness [12]. One review has indicated DP can directly improve mental health status of college students [13]. Research has also demonstrated the feasibility of using AI chatbots to deliver mental health support, with findings highlighting their effectiveness in alleviating anxiety and depressive symptoms [14]. Innovative approaches such as incorporating avatars and the metaverse into EMH services, have shown early promise but require further research evidence on effectiveness [15].

With the rapid technology development, EMH has gained much momentum and success [16]. Access to EMH can be many forms, such as videoconferences led by a professional, mental health mobile apps, or information and guidance on websites, with or without integration with electronic medical records (EMRs) [17]. It features prominently in delivering timely, effective mental health services by using technologies, at a low cost to reach a large population. Due to the anonymity it offers, EMH interventions also reduced help-seeking barriers, such as shame, stigma, and fear of exposure [18]. Advantages include improved accessibility in rural and remote areas and inner cities as well as multilingual tailor-made services that cater to the specific habits or preferences of users [19]. Use of internet-based mental health information and support is becoming increasingly common among the younger generations; for example over 60% Canadian youth were using it and over 80% prone to use a website if going through a difficult times [20]. The capacity of EMH to be delivered at scale provides an opportunity for the prevention of prevention of mental illness, as well as early detection and intervention [19].

## 3. Concerns over Data Safety in EMH 

Despite advantages and future trends of using EMH, data safety issues remain a significant concern [21]. Personal Health Information (PHI) such as name, age, and state of mental health are recorded and stored, [22] which is one of the main reasons for patients’ resistance to EMH services [23]. It is not uncommon to report digital mailbox hacks [24], text message interception [25], and video videoconference overheard or observed by unauthorized parties [26]. Nowadays, the growing wealth of mobile sensing data is being leveraged in health and behavioral sciences through digital biomarkers, aiding in the detection of mental health problems, monitoring progress, and enhancing targeted behavioral interventions [27, 28]. The integration of sophisticated sensors in smartphones and wearables enables the unobtrusive and automated collection of detailed, real-time data on human behaviors, states, and environmental factors [29, 30]. However, clients may unknowingly share various sensitive data with developers. In some cases, such data are sold to third parties for commercial purposes without notification or authorization, raising privacy concerns and mistrust from the public [31]. Previous analyses of mobile medical, health, and fitness apps has revealed privacy policies were completely lacking for 40% of paid apps; 40% of the apps collect highly traceable data including full name, health information, financial information, etc. Disturbingly, 83% of the free mobile health and fitness apps store data locally on the device without encryption [32]. According to the U.S. Department of Health and Human Services (HHS) Office for Civil Rights (OCR) data breach portal, approximately 295 breaches were reported by the healthcare sector in the first half of 2023 alone, with more than 39 million individuals implicated in healthcare data breaches [33]. Despite advancements in data encryption and safety protocols, the risk of data breaches cannot be entirely eliminated.

## 4. Current Well-known Data Protection Law and Regulations

It is encouraging to witness the significant efforts made by numerous countries in the realm of data protection. Table 1 lists some important regulations. These regulations typically include:

**Table 1.** **Important data protection regulations/enactments globally**.

Enactment date	Law/Regulation	Country/Region	Promulgating authority
1 July 1983	Privacy Act	Canada	The Parliament of Canada
13 April 2000	Personal Information Protection and Electronic Documents Act (PIPEDA)	Canada	The Parliament of Canada
12 December 1988	The Privacy Act 1988 (Privacy Act)	Australia	The Australian Parliament
21 August 1996	The Privacy Rule of the Health Insurance Portability and Accountability Act (HIPAA)	USA	The United States Congress
30 May 2003	Act on the Protection of Personal Information (APPI)	Japan	Japanese Government
15 October 2012	The Personal Data Protection Act (PDPA)	Singapore	Parliament of Singapore
25 May 2018	The European Union General Data Protection Regulation (EU GDPR)	European Union	European Parliament and Council of the European Union
25 May 2018	Data Protection Act 2018	UK	Parliament of the United Kingdom
13 September 2018	The California Consumer Privacy Act (CCPA)	USA	California State Legislature
20 August 2021	The Personal Information Protection Law (PIPL)	China	Standing Committee of the National People’s Congress
9 March 2022	The Personal Data Protection Act (PDPA)	Sri Lanka	The Parliament of Sri Lanka

(1) the necessity of obtaining user consent prior to collecting, using, and sharing personal information;

(2) data subject rights such as data access, portability and objection;

(3) accountability and obligations for data controllers/processors;

(4) oversight of cross-border data transfers or third-party sharing;

(5) data breaches reporting to affected parties;

(6) provisions for enforcement and penalties for non-compliance.

The General Data Protection Regulation (GDPR) in the EU is a comprehensive framework that sets a global benchmark for privacy laws. Its key features include:

(1) Standardized Communication: GDPR promotes the use of standardized icons and abbreviations to enhance user understanding of data collection processes.

(2) Informed Consent: It strengthens requirements for informed consent, mandating that data controllers and processors clearly explain the necessity of collecting health data, including legal or contractual obligations and potential consequences of non-disclosure.

(3) Data Management Guidelines: GDPR establishes stringent rules for the collection, storage, and transfer of personal data, ensuring robust data handling practices.

(4) Data Protection Impact Assessments (DPIAs): For high-risk processing activities, organizations are required to conduct DPIAs to identify and mitigate potential risks to individuals’ rights and freedoms. Breach Notification: Organizations must report data breaches to supervisory authorities within 72 hours, regardless of their scale, to promote transparency and accountability [34].

(5) Data Protection Officer (DPO): The regulation mandates the appointment of a DPO for many organizations, serving as an independent liaison between data subjects and authorities to ensure compliance [35].

(6) Severe Penalties: Non-compliance can result in hefty fines—up to 2% of global annual revenue or €10 million (USA $11.25 million) for minor breaches, and up to 4% or €20 million (USA $22.50 million) for major breaches—emphasizing the importance of adhering to GDPR standards [36].

Overall, GDPR not only enhances individual privacy rights but also inspires similar regulations globally, such as China’s PIPL and Singapore’s Personal Data Protection Act (PDPA).

## 5. Recommendations for Data Safety in the Future 

**Fig. 1.**
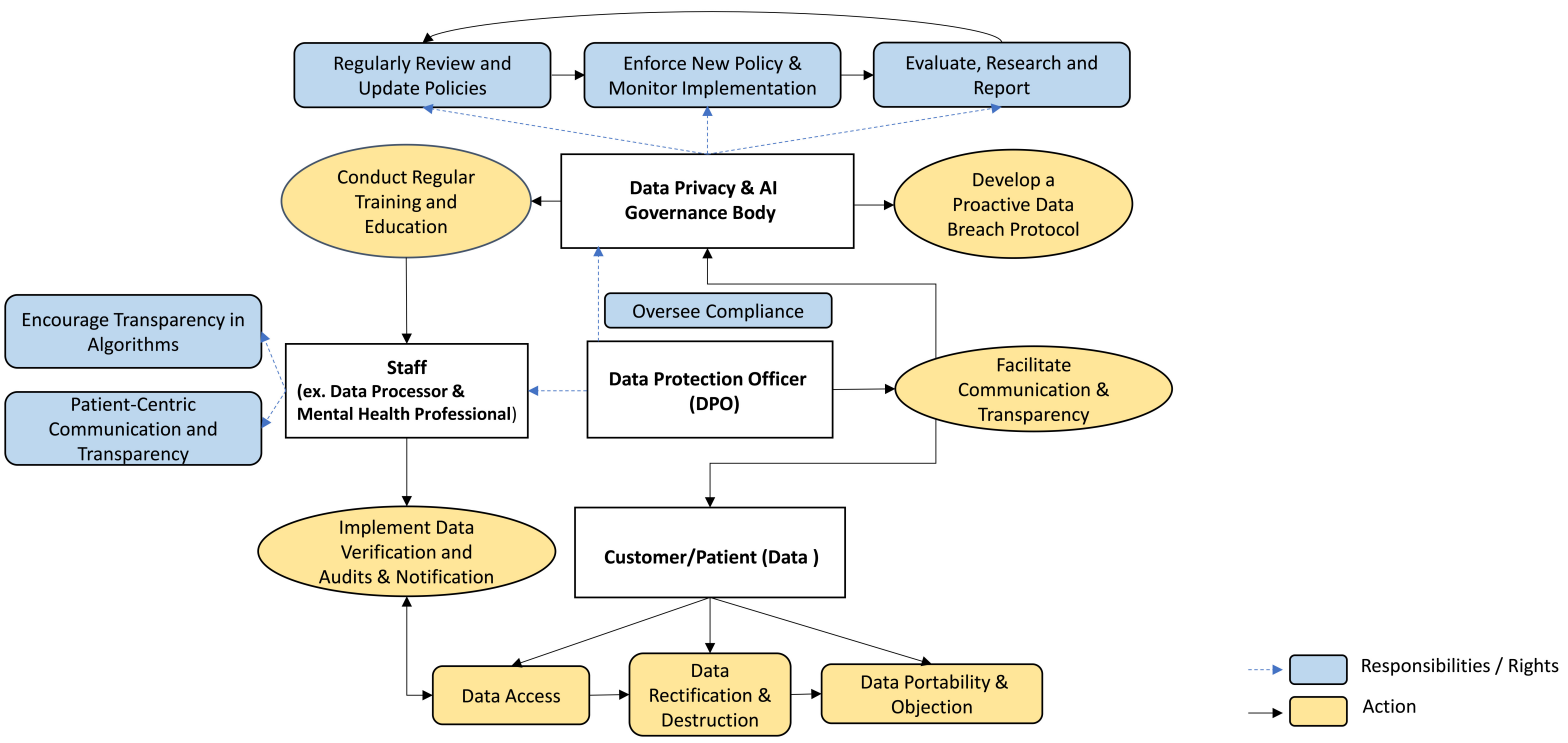
**Key recommendations for data safety governance**.

Data protection regulations and actions must evolve rapidly to keep pace with the evolving trend. Inconsistencies exist within different regulations. Some existing frameworks, like Health Insurance Portability and Accountability Act (HIPAA) (nearly 30 years old), are also criticized for failing to address data in digital health applications. EMH providers must immediately strengthen safeguards and implement robust privacy protocols. Key recommendations for essential future action are illustrated in Fig. 1 and outlined as follows.

### 5.1 Establish a Governance Body 

Create a dedicated governance body, to establish and regularly update privacy regulations. The governance body should ideally to be national, supplemented by sub-levels, and Companies are also suggested to have their own. It could be potentially integrated with AI oversight, for example under Hong Kong’s “artificial intelligence (AI) Model Personal Data Protection Framework” [37]. The body should also provide training and education, beside monitor regulation compliance and impact. Overall, it should facilitate cross-sector cooperation between government, industry, and healthcare stakeholders, and further provide insights for further actions and research.

### 5.2 Develop a Proactive Data Breach Protocol

Implement a proactive approach to privacy risk management, incorporating strategies from Privacy by Design (PbD) [38] and National Institute of Standards and Technology privacy framework (NIST) [39]. Along with a robust response plan for data breaches, it should establish clear protocols for identifying data processing roles, data types, and individual privacy needs. Beyond traditional privacy frameworks, consider implementing AI-powered systems that can dynamically identify, predict, and respond to potential data breaches or privacy risks. By utilizing machine learning models, these systems could analyse patterns in data access and usage to proactively flag unusual activities before they result in a breach.

### 5.3 Appoint a Data Protection Officer (DPO)

Designate a DPO to oversee compliance and transparency, facilitate clear communication between management and staff (such as mental health professionals), and address gaps in existing data protection plans. This role is critical for promoting a culture of accountability. The DPO should establish collaborative efforts across healthcare, technology, and regulatory sectors to develop unified standards for AI security in mental health care. These standards should focus on both the ethical use of AI and robust technical safeguards to prevent malicious AI manipulation or misuse.

### 5.4 Implement Data Verification and Audits

Ensure that patient consent, with terms easy to be understood, is prioritized throughout the data lifecycle. Establish verification procedures for data accuracy and conduct regular audits to maintain data integrity. Allow patients to update and modify their information, thereby enhancing autonomy and personalized mental health care.

### 5.5 Patient-Centric Communication and Transparency

Foster trust by maintaining open lines of communication with patients regarding data processing practices and respecting their rights to decline unwanted data transmission or access. Promote transparency in AI algorithms used in the care, allowing patients and stakeholders to understand how their data is utilized and the rationale behind AI-driven decisions. Keep patients informed about EMH care and solicit their feedback to align practices with their preferences and needs. Additionally, utilize blockchain technology to provide patients with full control over their data. Blockchain could facilitate secure, transparent, and immutable records of patient consent for data sharing, ensuring that users retain control over their mental health data at all times. This approach would enhance transparency and trust in digital mental health services and AI algorithms.

### 5.6 Provide User-friendly Procedures of Data Destruction

Furthermore, organizations should pay some attention to Ethical Implications of Data Destruction. Implement guidelines that balance patients’ right to delete their data with the clinical and legal responsibilities of healthcare providers. While patients should be able to erase data, this must not compromise the integrity of ongoing care, especially when the data is necessary for treatment or legal reasons. Legally, patients should be informed of their right to request the destruction of their data, and the systems managing mental health records should provide clear, user-friendly processes for data deletion requests.

### 5.7 Conduct Regular Training and Education

Mandate ongoing training for all stakeholders involved, especially data processors (e.g., IT staff) and mental health professionals, on data privacy and security, emphasizing the protection of PHI and recognizing security threats. This is crucial for building a knowledgeable workforce that prioritizes data protection. Additionally, educational activities should be conducted to enhance the public’s awareness and knowledge on this.

### 5.8 Regularly Review and Update Policies

Establish a framework for periodic review of data protection policies to adapt to emerging technologies and regulatory changes, ensuring ongoing compliance and relevance.

## 6. Conclusion 

Mental healthcare needs are rising rapidly. EMH has proven to be a transformative solution, with a promising future on the horizon. Tackling privacy concerns is a critical first step to propel EMH services to greater heights. However, it demands unwavering commitment from all relevant stakeholders to vouch for it. Collaborative efforts from all key stakeholders is essential. Now is the time to take decisive action to protect sensitive information and build trust in this vital service.
